# A Dominant-Negative FGF1 Mutant (the R50E Mutant) Suppresses Tumorigenesis and Angiogenesis

**DOI:** 10.1371/journal.pone.0057927

**Published:** 2013-02-28

**Authors:** Seiji Mori, Vu Tran, Kyoko Nishikawa, Teruya Kaneda, Yoshinosuke Hamada, Naomasa Kawaguchi, Masaaki Fujita, Yoko K. Takada, Nariaki Matsuura, Min Zhao, Yoshikazu Takada

**Affiliations:** 1 Department of Molecular Pathology, Osaka University Graduate School of Medicine, Division of Health Sciences, Suita, Osaka, Japan; 2 Department of Dermatology, School of Medicine, University of California Davis, Sacramento, California, United States of America; 3 Department of Biochemistry and Molecular Medicine, School of Medicine, University of California Davis, Sacramento, California, United States of America; IDI, Istituto Dermopatico dell’Immacolata, Italy

## Abstract

Fibroblast growth factor-1 (FGF1) and FGF2 play a critical role in angiogenesis, a formation of new blood vessels from existing blood vessels. Integrins are critically involved in FGF signaling through crosstalk. We previously reported that FGF1 directly binds to integrin αvβ3 and induces FGF receptor-1 (FGFR1)-FGF1-integrin αvβ3 ternary complex. We previously generated an integrin binding defective FGF1 mutant (Arg-50 to Glu, R50E). R50E is defective in inducing ternary complex formation, cell proliferation, and cell migration, and suppresses FGF signaling induced by WT FGF1 (a dominant-negative effect) *in vitro.* These findings suggest that FGFR and αvβ3 crosstalk through direct integrin binding to FGF, and that R50E acts as an antagonist to FGFR. We studied if R50E suppresses tumorigenesis and angiogenesis. Here we describe that R50E suppressed tumor growth in vivo while WT FGF1 enhanced it using cancer cells that stably express WT FGF1 or R50E. Since R50E did not affect proliferation of cancer cells *in vitro*, we hypothesized that R50E suppressed tumorigenesis indirectly through suppressing angiogenesis. We thus studied the effect of R50E on angiogenesis in several angiogenesis models. We found that excess R50E suppressed FGF1-induced migration and tube formation of endothelial cells, FGF1-induced angiogenesis in matrigel plug assays, and the outgrowth of cells in aorta ring assays. Excess R50E suppressed FGF1-induced angiogenesis in chick embryo chorioallantoic membrane (CAM) assays. Interestingly, excess R50E suppressed FGF2-induced angiogenesis in CAM assays as well, suggesting that R50E may uniquely suppress signaling from other members of the FGF family. Taken together, our results suggest that R50E suppresses angiogenesis induced by FGF1 or FGF2, and thereby indirectly suppresses tumorigenesis, in addition to its possible direct effect on tumor cell proliferation *in vivo.* We propose that R50E has potential as an anti-cancer and anti-angiogenesis therapeutic agent (“FGF1 decoy”).

## Introduction

The FGF family consists of 22 related polypeptides that are expressed in almost all tissues and are multifunctional. They can be subdivided in canonical (cFGFs, FGF7-10, FGF16-20, FGF22), intracellular (iFGFs, FGF11-14), and hormonelike (hFGFs, FGF19, 21 and 23) subfamilies [1]. Some FGFs, like FGF1 and FGF2, have potent angiogenic activity and are implicated as promoters of angiogenesis, the formation of new blood vessels, in cancer and chronic inflammatory diseases. FGFs also increase the motility and invasiveness of a variety of cell types [2]–[4]. The biological effects of FGFs are mediated by four structurally related receptor tyrosine kinases: FGFR1, FGFR2, FGFR3, and FGFR4. The binding of FGF to its receptor results in receptor dimerization and subsequent transphosphorylation of specific tyrosine residues within the cytoplasmic domain. This leads to the activation of intracellular signaling cascades. The four main signaling pathways downstream of receptor activation are 1) the Janus kinase/signal transducer and activator of transcription (Jak/Stat), 2) phosphoinositide phospholipase C (PLC**γ**), 3) phosphatidylinositol 3-kinase (PI3K), and 4) mitogen-activated protein kinase/extracellular signal-regulated kinase (MAPK/Erk). [2]–[4]. FGF1 binds to all known cell-surface FGFR isoforms (FGFR1b, 1c, 2b, 2c, 3b, 3c, and 4) [2]–[4].

FGFs are potent mitogens for many cancer cells. More than 80% of prostate cancer cells express FGF8, and the levels of FGF8 expression correlate with the levels of invasiveness [5]. In breast cancer cells, cells that overexpress FGF1 or FGF4 grow faster than cells with low FGF expression in vivo [6]. The levels of FGFR expression also correlate with the invasiveness of cancer [7]. FGF1/FGFR1 signaling (both autocrine and paracrine loops) thus plays a critical role in cancer progression. Because FGF signaling enhances multiple biological processes that promote tumor progression, it is an attractive therapeutic target, particularly since therapies targeting FGF receptors and/or FGF signaling may affect both the growth of tumor cells and angiogenesis. FGF plays a role in pathological angiogenesis in inflammatory diseases. Transient exposure to FGF1 upregulates the expression of the cell adhesion molecules ICAM (intercellular adhesion molecule)-1 and VCAM (vascular cell adhesion molecule)-1 in endothelial cells and increases polymorphonuclear leukocyte adhesion and transendothelial migration [8].

Integrins are a family of cell adhesion receptors that recognize extracellular matrix ligands and cell surface ligands [9]. Integrins are transmembrane α−β heterodimers, and at least 18 α and 8 β subunits are known [10]. Integrins are involved in signal transduction upon ligand binding and their functions are in turn regulated by signals from within the cell [10]. Crosstalk between integrins and growth factor receptors are an important signaling mechanism during normal development and pathological processes [11]. We previously reported that FGFR and integrins crosstalk through direct integrin binding to FGF [12]. We first predicted that FGF1 binds to integrin αvβ3 using docking simulation. We found that FGF1 directly binds to integrin αvβ3 (KD about 1 µM) [12]. Antagonists to αvβ3 (mAb 7E3 and cyclic RGDfV) block this interaction. The CYDMKTTC sequence (the specificity loop) within the ligand-binding site of β3 plays a role in FGF1 binding, suggesting that FGF1 binds to a binding site common to other αvβ3 ligands. The integrin binding site in FGF1 is distinct from the FGFR-binding site. We identified an FGF1 mutant (R50E) that is defective in integrin binding but still binds to heparin and FGFR. R50E is defective in inducing DNA synthesis, cell proliferation, cell migration, and chemotaxis, suggesting that the direct integrin binding to FGF1 is critical for FGF signaling. WT FGF1 induces both transient (within 3 hours of stimulation) and sustained activation of ERK1/2 (after 3 hours of stimulation) in NIH3T3 cells. In contrast, R50E is defective in inducing sustained ERK1/2 activation while it induces transient ERK1/2 activation. R50E induces transient activation but is defective in sustained activation of FGFR1 and FRS2α as well [13]. Importantly, WT FGF1 induces ternary complex formation (integrin-FGF-FGFR1) but R50E is defective in this function [13]. We propose a model in which integrin and FGFR bind to FGF1 simultaneously and make a ternary complex on the cell surface. Our model predicts that the R50E mutant should compete with WT FGF1 for binding to integrins. Thus, R50E should be antagonistic. We discovered that R50E is actually a dominant-negative mutant of FGF1 in vitro. Excess R50E suppresses DNA synthesis and cell proliferation induced by WT FGF1 [13].

Taken together, our previous results suggest that 1) Ternary complex formation is involved in FGF signaling, 2) the defect of R50E to bind to integrin may be directly related to the functional defective of R50E, and 3) R50E is a dominant-negative mutant. These results suggest that R50E has potential as a therapeutic in cancer [13]. These results suggest that R50E has translational potential: R50E can be an anti-angiogenesis and anti-cancer therapeutic. To address this hypothesis, in the present study, we studied the effect of R50E on angiogenesis and tumorigenesis.

## Materials and Methods

All chemicals were purchased from Sigma (St. Louis, MO) or Nacalai tesque (Kyoto, Japan) unless otherwise stated. Wild-type FGF (WT) and mutant form FGF (R50E) were bacterially expressed and purified as described previously [12]. HRP-conjugated anti-His tag antibody was purchased from Qiagen (Valencia, CA). Human umbilical endothelial cells (HUVEC) were purchased from Sanko-junyaku (Tokyo, Japan) and were routinely cultured in EGM-2 Bullet kit (Lonza Basel, Switzerland) supplemented with 2% FCS. DLD-1 human colon carcinoma cells were obtained from American Type Culture Collection (ATCC) and were maintained in RPMI1640 supplemented with 10% FCS and antibiotics.

### Generation of DLD-1 Colon Carcinoma Cells that Secrete WT or R50E FGF1 and Tumorigenesis in vivo

We inserted the 6-His and S tags in the Kpn I/Bam HI site in pSecTag vector as described [14] and inserted WT or mutant FGF1 cDNA fragment (Bgl II/Bam HI fragment) into the Bam HI site of the vector. We transfected the pSecTag construct encoding WT or mutant FGF1 to DLD-1 cells, and selected for zeocin resistance. We detected the secretion of WT and R50E mutant in DLD-1 cells by concentrating the culture medium (15X) using ulrafiltration and by Western blotting with HRP-labeled anti-6His antibodies. These cells were subcutaneously injected into nude mice (10^6^ cells/mouse) without further cloning or enrichment. The tumor growth was monitored using caliper, and tumor volume (v) was calculated as described [15].

### Cell Migration

A polycarbonate filter of 8 µm pore size of the transwell insert was used to test cell Migration. Lower side of the filter was coated with 10 µg/ml fibronectin (Asahi Glass, Tokyo) for 1 h at room temperature. After washing, the insert was placed into a 24-well cell culture plate, and the lower portion of the plate was filled with 600 µl of serum-free EBM-2 medium containing 5 ng/ml WT FGF1 or the mixture of WT FGF1 (5 ng/ml) and R50E (250 ng/ml) in the presence of 5 µg/ml heparin. HUVEC cells (6×10^4^ cells/filter) were plated on the filter and incubated at 37°C for 6 h, and cells were visualized by crystal violet staining. The uncoated upper side of each filter was wiped with a cotton swab to remove cells that had not migrated through the filter. Chemotaxed cells were counted from the digital images of the stained cells. Results are expressed as means ± S.E. of the cell number.

### Endothelial Cell Tube Formation

Serum starved HUVECs were plated in wells (3×10^4^ cells/well) of 48 well plate coated with 150 µl Matrigel (BD Biosciences, San Jose, CA) in serum-free EBM-2 medium. The medium contains 5 ng/ml WT FGF1, or the mixture of WT FGF1 (5 ng/ml) and R50E (250 ng/ml) in the presence of 5 µg/ml heparin. Cells were incubated for 8 h at 37°C. Images were observed under Nikon Eclipse TE2000E inverted microscope with 4× objective lens (Nikon). The Number of vessel branch points of tube per field was counted from the digital images. Results are expressed as means ± S.E. of the numbers of vessel branch points.

### Matrigel Plug Assay

Matrigel plugs containing 1 µg/ml FGF-WT, 1 µg/ml FGF-R50E, or the mixture of 1 µg/ml WT FGF1 and 50 µg/ml FGF-R50E were prepared on ice. The plugs (1 ml each) were injected subcutaneously into the back of 12 weeks old SD rat. The matrigel plugs were removed 10 days after injection, fixed with formalin, and embedded in paraffin block. Tissue sections were stained with antibodies against von Willebrand factor (Dako Glostrup, Denmark), a blood vessel marker. The number of blood vessels was counted in 5 independent areas of a section under a light microscope. Results are expressed as means ± S.E. of the stained cell number.

### Rat Aorta Ring Assay

Culturing of aortic explants in three-dimensional collagen gel was performed as described [16]. Briefly, thoracic aortas were removed from 6 weeks old Sprague-Dawley (SD) rat. The periaortic fibroadipose tissue was carefully removed and sectioned at approximately 1 mm thickness. Cellmatrix porcine type I collagen (3 mg/ml) (Nitta gelatin) was gelled in 24 well plate at 37°C for 30 min. Ring shaped aortas were embedded in the gels and immersed in medium containing 50 ng/ml WT FGF1, 50 ng/ml R50E, or the mixture of WT and R50E (50 ng/ml and 2.5 µg/ml) and incubated at 37°C for 10 days. Media were changed every day. The spatial distributions of microvessel sprouts were observed using phase-contrast inverted microscope with digital camera.

### Chick Embryo Chorioallantoic Membrane (CAM) Assay

CAM assays were performed as previously described [17], [18]. Briefly, fertilized chick eggs were grown in a rotating humidified incubator for 11 days until blood vessels fully developed. We then created a window in the eggshell to expose the underlying chorioallantoic membrane. After securing the eggs in the horizontal position, we placed 6 mm filter discs filled with saline or saline+FGF directly over a vessel within the membrane. The eggs were incubated for another 2 days. At day 13 we excised the membrane surrounding the filter and captured a digital image (using MoticImage software) to count the total number of vessel branch points directly beneath the disc.

### Other Method

MTS assays were performed as described [19].

### Statistical Analysis

Statistical analysis was performed using Prism software (GraphPad software).

### Ethics Statement

This study was carried out in strict accordance with the recommendations in the Guide for the Care and Use of Laboratory Animals of the National Institutes of Health, University of California Davis, and Osaka University. Protocols were approved by University of California Davis Institutional Animal Care and Use Committee and the Animal Experiment Committee of Osaka University.

## Results

### Suppression of Tumorigenesis in vivo by R50E

We have reported that FGF1 specifically binds to integrin αvβ3 [12]. The FGF1 mutant (R50E) is defective in integrin binding but still binds to heparin and FGFR. R50E is defective in inducing DNA synthesis, cell proliferation, cell migration, and chemotaxis, suggesting that the direct integrin binding to FGF1 is critical for FGF signaling [12]. WT FGF1 induces ternary complex formation (integrin-FGF1-FGFR1) in NIH3T3 cells and human umbilical endothelial cells (HUVECs), but R50E is defective in these functions. WT FGF1 induces sustained activation of ERK1/2, but R50E is defective in this function. Notably excess R50E suppresses signals induced by WT FGF1 in vitro. Our results suggest that 1) R50E is a dominant-negative mutant, 2) ternary complex formation is involved in FGF signaling, and 3) the defect of R50E to bind to integrin may be directly related to the antagonistic action of R50E. Taken together, these results suggest that R50E has potential as a therapeutic in cancer [13].

**Figure 1:**
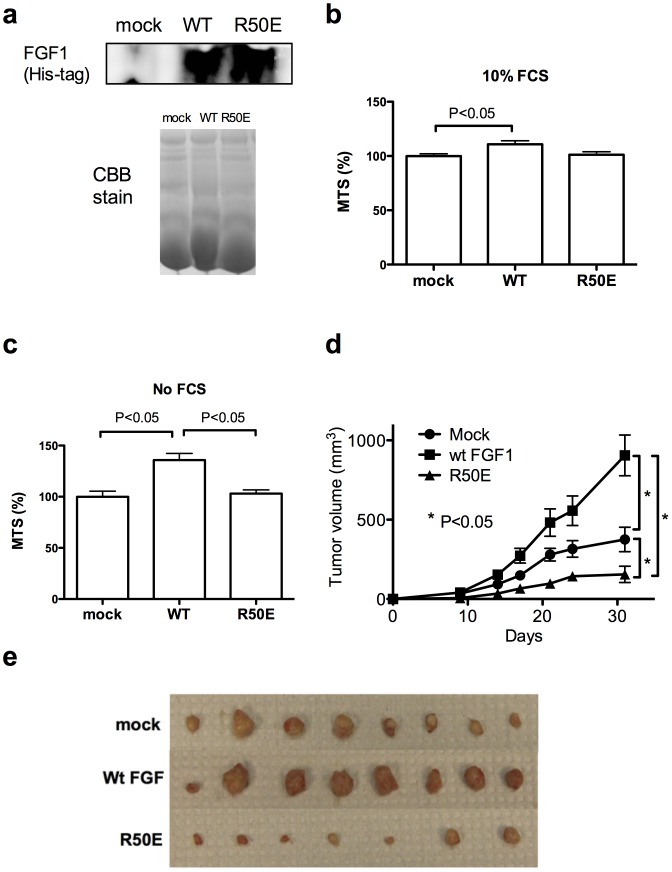
R50E suppresses tumorigenesis in vivo. a. Transfected DLD-1 cells secrete WT FGF1 or R50E into culture medium. DLD-1 cells that stably express WT FGF1 or R50E were generated. The WT FGF1 and R50E have a 6His-tag at the N-terminus. To detect FGF1 secreted from the transfected cells, we analyzed the culture media by Western blotting with anti-6His antibodies. Mock-transfected cells were used as a control. As a loading control, we ran the same samples in gel in parallel and stained the gel with Coomassie Brilliant Blue (CBB). b. Proliferation of DLD-1 cells in the presence of 10% FCS. DLD-1 cells that secrete R50E grew in the medium that contains FCS in vitro at levels comparable to those of WT-FGF1 expressing cells or mock transfected cells. Statistical analysis was done by one-way ANOVA plus Tukey analysis. c. Proliferation of DLD-1 cells in the absence of FCS. DLD-1 cells that secrete R50E grew in vitro in the medium without FCS at levels comparable to that of mock-transfected cells. Cells that express WT FGF1 grew faster than mock-transfected and R50E expressing cells. Statistical analysis was done by one-way ANOVA plus Tukey analysis. d. The growth curve of DLD-1 cells in vivo. WT FGF1 enhanced tumor growth in vivo, while R50E suppressed it (as shown by the growth curve and the sizes of DLD-1 tumors removed at day 31). We injected the DLD-1 cells that secrete WT FGF1or R50E into nude mice (1 million cells/site) at right and left inguinal regions (4 mice per group, 2 tumors/mouse). Mock-transfected cells were used as a control. Statistical analysis of tumor sizes at Day 31 was done by t-test (n = 8 for mock and wt FGF, n = 7 for R50E). e. The sizes of tumors at Day 31. DLD-1 cells secreting wt FGF1 grew faster, and cells secreting R50E slower, than mock-transfected cells (n = 8 for mock and wt FGF, n = 7 for R50E).

To test if R50E may act as an antagonist to FGF signaling in vivo, we stably expressed R50E or WT FGF1 in a secretion vector in DLD-1 colon carcinoma cells, and tested if R50E affects tumor growth in vivo. These cells secreted 6His-tagged R50E or WT FGF1 into culture medium (Fig. 1a). The expression of WT FGF1 or R50E had little or no effect on cell survival in vitro in the presence of FCS (Fig. 1b). The expression of WT FGF1 significantly enhanced cell survival in the absence of serum, but the expression of R50E did not (Fig. 1c). When the population of DLD-1 colon cancer cells that stably express WT FGF1 or R50E were injected subcutaneously into nude mice (1 million cells/site, two sites per mouse), cells that secrete WT FGF1 generated bigger tumors (n = 8) but cells that secrete R50E generated smaller tumors (n = 8) than mock-transfected cells (n = 7) (Fig. 1d and 1e). These results suggest that R50E suppressed tumorigenesis in vivo while WT FGF1 markedly enhanced it. Since R50E did not affect tumor cell proliferation or survival in vitro, it is likely that R50E suppressed tumorigenesis in vivo indirectly through blocking FGF signaling in endothelial cells (angiogenesis) or stromal cells. We thus tested the effect of R50E on angiogenesis.

### R50E Suppresses WT FGF-1 Induced Endothelial Cell Migration

**Figure 2:**
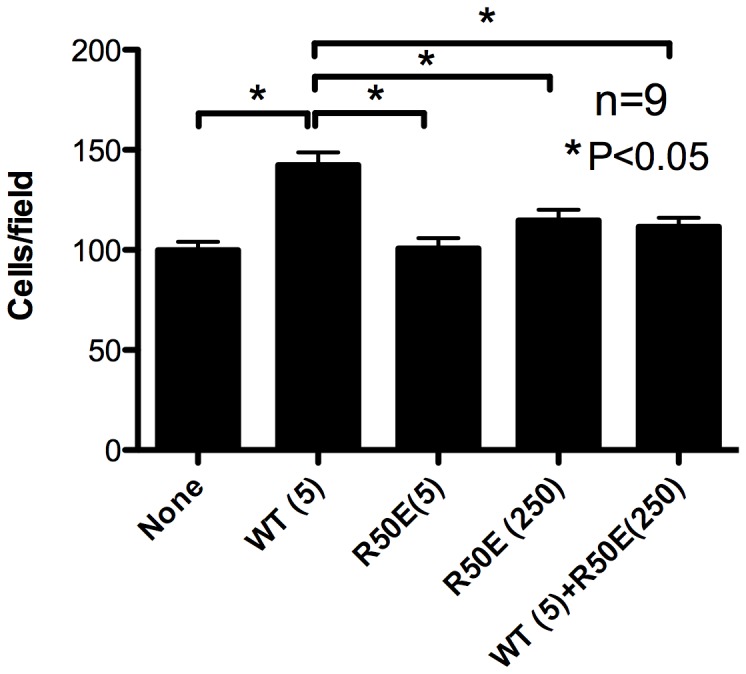
R50E suppresses WT FGF1-induced endothelial cell migration. Lower side of the filter in the modified Boyden chamber was coated with fibronectin (10 µg/ml). The lower chamber was filled with serum-free medium with WT FGF1 (5 ng/ml) or the mixture of WT FGF1 and excess R50E (5 and 250 ng/ml, respectively). HUVECs were plated on the filter and incubated for 6 h. Chemotaxed cells were counted from the digital images of the stained cells. Data is shown as means +/− SE per field. Statistical analysis was done by one-way ANOVA plus Tukey analysis.

Endothelial cell migration is a critical feature of tumor angiogenesis. We tested the effect of R50E on migration of HUVECs. Lower side of the filter in the modified Boyden chamber was coated with fibronectin (10 µg/ml). The lower chamber was filled with serum-free EBM-2 medium with WT FGF1 (5 ng/ml) and/or R50E (5 and 250 ng/ml, respectively). HUVECs were plated on the filter and incubated for 6 h, and cells were stained with crystal violet. Chemotaxed cells were counted from the digital images of the stained cells. We found that R50E did not induce cell migration at 5 and 250 ng/ml concentration (Fig. 2). Excess R50E significantly suppressed migration of HUVECs induced by WT FGF1 (Fig. 2). This suggests that R50E acts as an antagonist of FGF1 in migration of HUVECs.

### R50E Suppresses WT FGF1 Induced Tube Formation of Endothelial Cells

**Figure 3:**
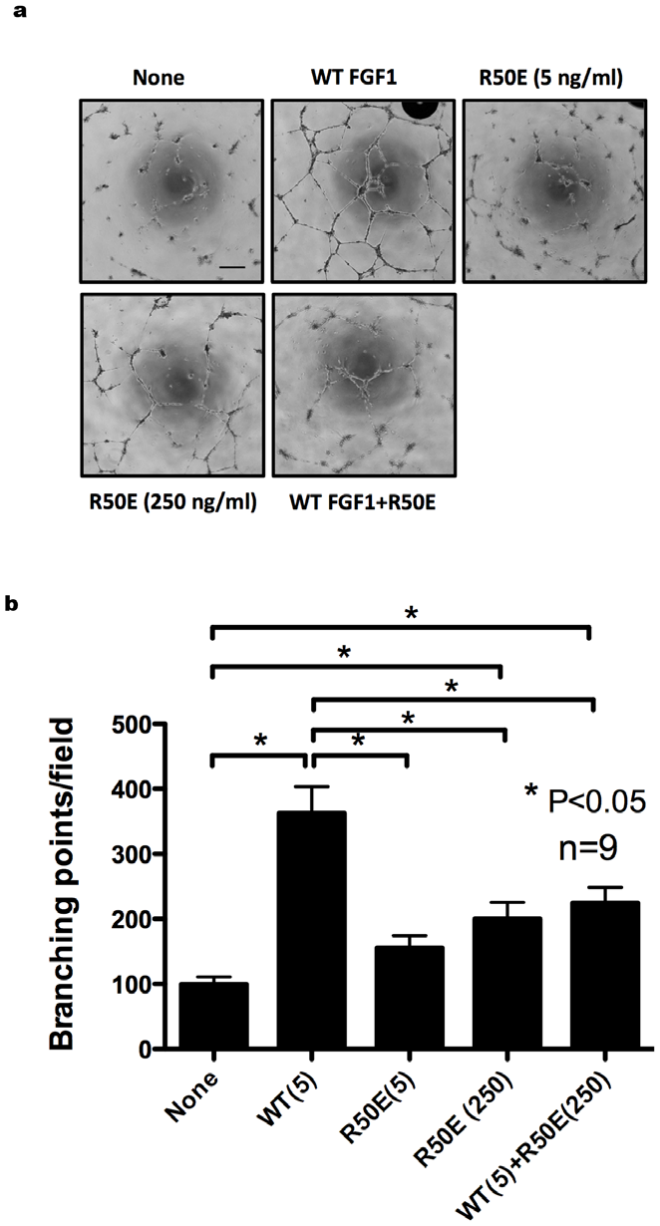
R50E suppresses WT FGF1- induced tube formation of endothelial cells in vitro. Serum starved HUVECs were plated on Matrigel-coated plates, and incubated in WT FGF1 (5 ng/ml) or the mixture of WT FGF1 (5 ng/ml) and R50E (250 ng/ml) for 8 h. a. Representative tube formation images are shown. Scale bar = 200 µm. b. The number of branch points was counted per field from the digital images. Data is shown as means +/− SE. Statistical analysis was done by one-way ANOVA plus Tukey analysis.

One of the most specific tests for angiogenesis is the measurement of the ability of endothelial cells to form three-dimensional structures (tube formation) [20]. Endothelial cells of all origins appear to be able to form tubules in vitro on extracellular matrix components. We examined the effect of R50E on the tube formation of HUVECs in vitro. We plated serum-starved HUVECs on reconstituted extracellular matrix (Matrigel, growth factor reduced)-coated plates, and incubated with WT FGF1 and/or R50E (5 and 250 ng/ml, respectively) for 8 h. We counted the number of branching points per field from the digital images. We found that WT FGF1 markedly enhanced tube formation and R50E (5 ng/ml) did not induce tube formation. High dose R50E weakly induced tube formation. Excess R50E (250 ng/ml) significantly suppressed tube formation induced by WT FGF1 (Fig. 3). This suggests that R50E directly affects endothelial cell and competes with WT FGF1 for its binding to integrin to generate tube-like structure.

### R50E Suppresses Angiogenesis in the Rat Aorta Ring Assays

**Figure 4:**
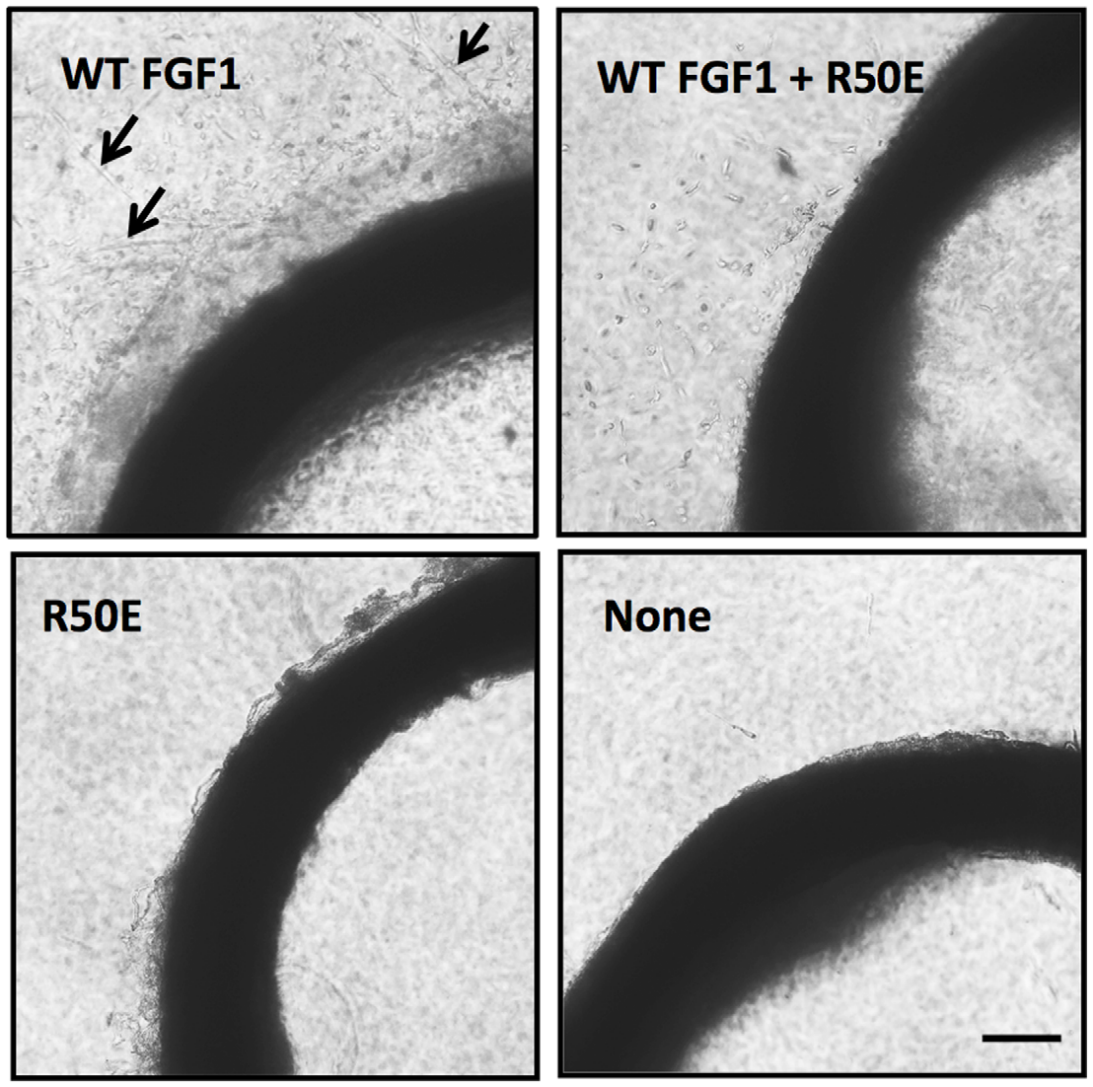
R50E suppresses WT FGF1-induced angiogenesis in rat aortic ring. Isolated rat aortic ring was embedded in collagen gels in DMEM containing WT FGF1 (50 ng/ml), R50E (50 ng/ml) or the mixture of WT FGF1 (50 ng/ml) and R50E (2500 ng/ml) and cultured for 10 days. Representative phase contrast images of 3 independent experiments are shown. Scale bars, 100 µm.

To test the effect of R50E on angiogenesis in more physiological conditions, we performed an aorta ring assay. This organ culture assay uniquely recapitulates the key steps in the process such as matrix degradation, migration, proliferation, and reorganization while other in vitro assays are designed to study a particular step in the angiogenesis. Isolated rat aortic ring was embedded in collagen gels in DMEM containing WT FGF1, R50E, or the mixture of WT FGF1 and excess R50E and cultured for 10 days. WT FGF1 (50 ng/ml) markedly induced the outgrowth of cells from aortic arch, but R50E (50 ng/ml) did not (Fig. 4). Excess R50E (2500 ng/ml) significantly suppressed the outgrowth of cells induced by WT FGF1 (50 ng/ml). This indicates that R50E suppresses newly sprouting vessels induced by WT FGF1.

### R50E Suppresses Angiogenesis in Matrigel Plug Assays

**Figure 5:**
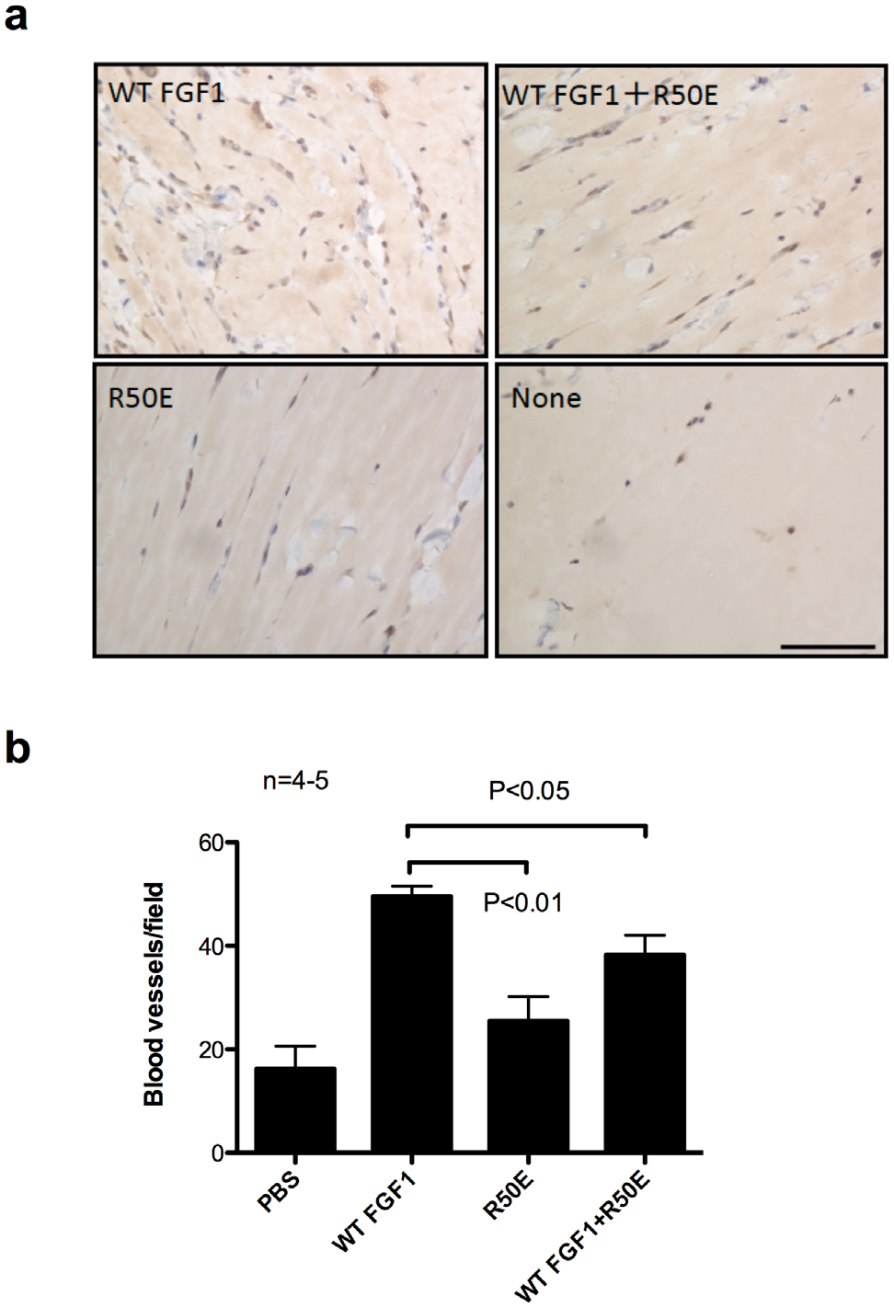
R50E suppresses angiogenesis in Matrigel plug assays in rat. Matrigel plug containing WT FGF1 (1 µg/ml), R50E (1 µg/ml) or the mixture of WT FGF1 (1 µg/ml) and excess R50E (50 µg/ml) were injected subcutaneously into the back of rat, respectively. The plugs (n = 4−5) were removed 10 days after injection and tissue sections were stained for von Willebrand factor, a blood vessel marker. a. Representative images are shown. Scale bar = 50 µm. b. The number of extended blood vessels were counted under a light microscope. Data is shown as means +/− SE. Statistical analysis was done by one-way ANOVA plus Tukey analysis.

The evaluation of angiogenesis influencing factors is ultimately best made in vivo. We asked whether R50E is capable of inhibiting WT FGF1 induced angiogenesis in vivo in a matrigel plug assay. We injected matrigel plugs that contain WT FGF1 (1 µg/ml), R50E (1 µg/ml), or the mixture of WT FGF1 (1 µg/ml) and excess R50E (50 µg/ml) subcutaneously into the back of rat. We removed the plugs 10 days after injection and determined the levels of angiogenesis by staining tissue sections for von Willebrand factor, a marker for blood vessels. The number of extended blood vessels was counted. We found that WT FGF1 markedly increased the number of blood vessels, whereas R50E was defective in this function (Fig. 5). Excess R50E reduced the number of blood vessels induced by WT FGF1. These findings suggest that R50E suppresses angiogenesis induced by WT FGF1 in vivo.

### R50E Suppresses Angiogenesis in Chick Embryo Chorioallantoic Membrane (CAM) Model

**Figure 6:**
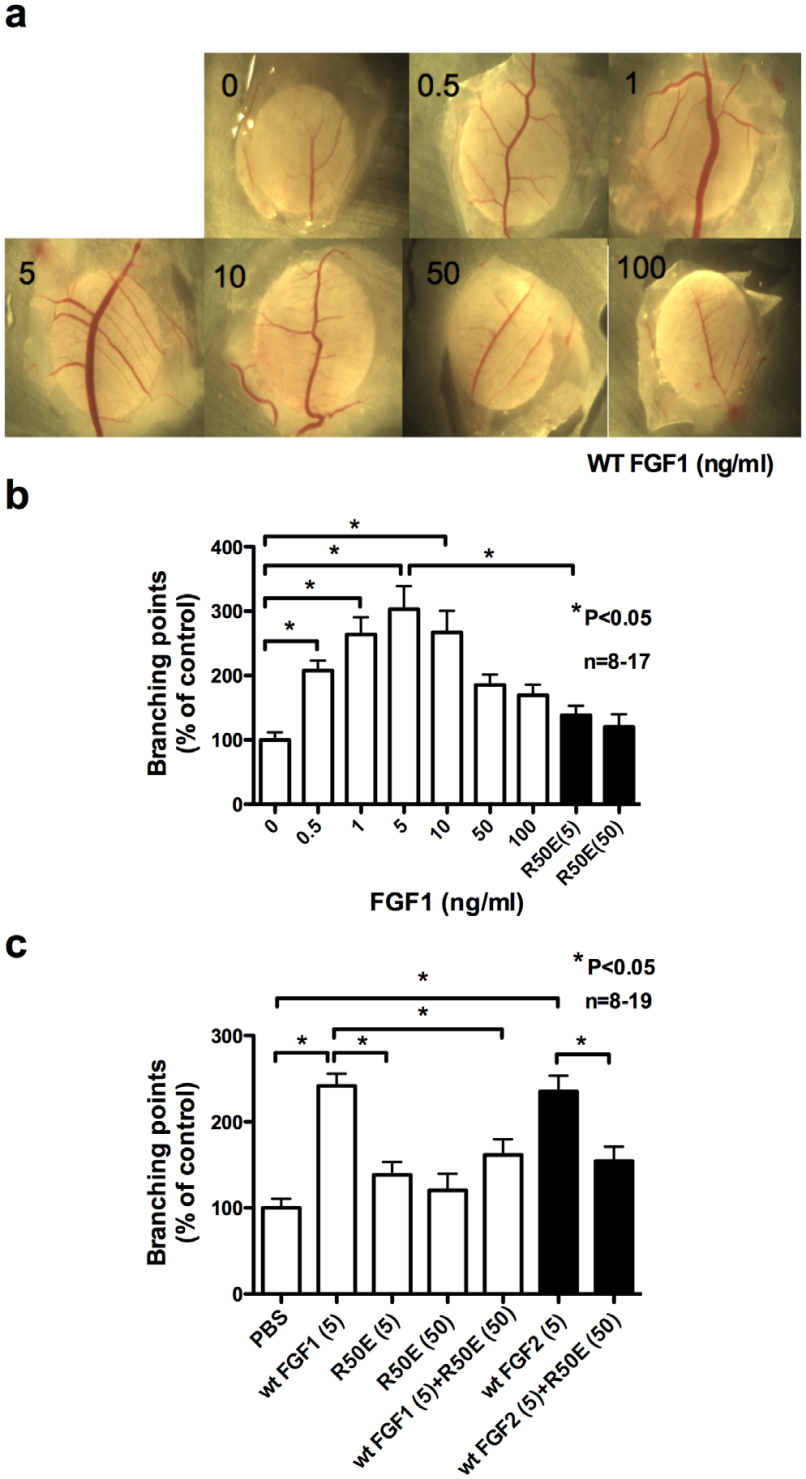
R50E suppresses FGF1- and FGF2-induced angiogenesis (branching formation) in CAM models. Saline- or FGF- impregnated filter disks are placed on blood vessels in otherwise avascular sections of CAM (day 11) to induce angiogenesis. The disks and underlying CAM tissue (day 13) are then harvested. Neovascularization was then scored by counting vessel branches present in the CAM tissue below the filter from digital images. a) and b) Quantification of dose response. Five ng/ml is optimum, c) Suppression of FGF1-induced angiogenesis by excess R50E. d) Suppression of FGF2-induced angiogenesis by excess R50E. The data suggest that R50E suppresses FGF1- and FGF2-induced angiogenesis in the CAM model. Statistical analysis was done by one-way ANOVA plus Tukey analysis.

CAM is another widely utilized in vivo system to study angiogenesis and anti-angiogenesis and it is easier to quantify angiogenesis in this assay than other assays. We placed saline- or FGF- impregnated filter disks on blood vessels in avascular sections of CAM (day 11) for 48 h to induce angiogenesis. The disks and underlying CAM tissue (day 13) were then harvested. We scored angiogenesis by counting vessel branches present in the CAM tissue below the filter from digital images. We first determined optimum dose of wt FGF1 for angiogenesis (Fig. 6a, 6b). Five ng/ml of wt FGF1 was optimum. R50E (5 and 50 ng/ml) did not induce angiogenesis. We tested if excess R50E (50 ng/ml) suppresses angiogenesis induced by WT FGF1 (5 ng/ml). Notably, excess R50E suppressed angiogenesis induced by WT FGF1 (Fig. 6c). This suggests that R50E shows an anti-angiogenic action in this model as well. Since FGF1 binds to all known FGFRs (FGFR1-4), R50E is expected to suppress FGFR signaling induced by other members of the FGF family. We tested if R50E suppresses angiogenesis induced by FGF2. We found that this is the case: excess R50E suppressed angiogenesis induced by WT FGF2 (Fig. 6c). The data suggest that R50E suppresses FGF1- and FGF2-induced angiogenesis in the CAM model.

Taken together, R50E was defective in inducing angiogenesis, and effectively suppressed angiogenesis in different in vitro and in vivo angiogenesis models. It is likely that R50E may indirectly suppress tumorigenesis in vivo through suppressing angiogenesis.

## Discussion

### R50E is an Anti-angiogenic Agent

In the present study, we establish that R50E suppressed tumor growth in vivo while WT FGF1 enhanced it using cancer cells that stably express WT FGF1 or R50E. Since R50E showed little or no effect on proliferation of cancer cells in vitro, we hypothesized that R50E indirectly suppressed tumorigenesis through suppressing angiogenesis. Excess R50E suppressed migration and tube formation of HUVEC, and suppressed angiogenesis in aorta ring assays and matrigel plug assays, suppressed angiogenesis in chick embryo chorioallantoic membrane (CAM) assays, which is induced by WT FGF1. Taken together, our results suggest that R50E suppresses angiogenesis induced by FGF1 and thereby may indirectly suppress tumorigenesis, in addition to its possible direct effect on tumor cell proliferation in vivo. Furthermore, excess R50E suppressed FGF2-induced angiogenesis in CAM assays, suggesting that R50E may uniquely suppress signaling from other members of the FGF family. We propose that R50E has potential as an anti-cancer and anti-angiogenesis therapeutic agent (“FGF1 decoy”).

### Potential Advantage of R50E Over Antibodies and Kinase Inhibitors

Potential advantage of the FGF1 mutant R50E is that 1) R50E is highly specific to FGFR1 compared to tyrosine kinase inhibitors, which are selective rather than specific, and 2) R50E may have higher affinity to FGFR1 (KD 10^−12^ M) than antibodies to FGFR1 (KD 10^−7^ to 10^−11^ M). Thus, we expect that much lower dose may be required than antibodies to FGFR1. Also, 3) the large size of antibodies results in poor tissue penetration [21], whereas R50E could more fully interrogate a tumor mass. And 4) Currently used target therapeutics (antibodies and kinase inhibitors) almost always induce resistance after a while. This is partly due to point mutations in antibody epitopes or inhibitor-binding sites. Cancer cells obviously benefit from mutations that block the binding of antagonists. We believe that R50E may not induce such mutations in FGFR because R50E and FGF1 bind to FGFR exactly the same way, and blocking binding of FGF1 (and other members of the FGF family) to FGFR would not benefit cancer cells.

### Can we Use Mutant Proteins as Therapeutics?

There is a precedent that a mutant of human protein was used for human diseases. A mutant of human growth hormone (hGH) has been used as an antagonist of GH receptor in the treatment of acromegaly (Pegvisomant) [22]. The Gly-120 of hGH was mutated to Arg (G120R) and this mutant was further modified by poly(ethylene glycol) (PEG)-5000 to elongate half-life. Pegvisomant prevents functional dimerization of hGH receptor by sterically inhibiting conformational changes within the GHR dimers [22]. Pegvisomant is generally well tolerated with a safety profile similar to that reported in clinical trials and can effectively reduce IGF1 in patients with acromegaly refractory to conventional therapy [23].

We need to fully evaluate the potential of R50E as a therapeutic agent in future studies. However, it is expected that R50E protein may have a short half-life, and it may be rapidly cleared from circulation. We will need to stabilize R50E and deliver it to the tumor area to effectively suppress angiogenesis and tumorigenesis in vivo. Interestingly, we discovered that direct integrin growth factor interaction is also important for IGF1 [24], [25] and NRG1 [19]. We propose that integrin-growth factor receptor crosstalk through direct integrin-binding to growth factor and subsequent ternary complex formation may be a common mechanism for the crosstalk and integrin-growth factor interaction may be a novel therapeutic target.
