# Correction: A Dominant-Negative FGF1 Mutant (the R50E Mutant) Suppresses Tumorigenesis and Angiogenesis

**DOI:** 10.1371/journal.pone.0091599

**Published:** 2014-02-28

**Authors:** The PLOS ONE Staff

Jun Saegusa was incorrectly omitted from the list of authors of the article. Dr. Saegusa should be indicated as the eighth author and affiliated with Department of Dermatology, School of Medicine, University of California Davis, Sacramento, California, United States of America. The contributions of this author are as follows: Performed the experiments and analyzed the data.
